# The impact of coronavirus disease 2019 on emotional and behavioral stress of informal family caregivers of individuals with stroke or traumatic brain injury at chronic phase living in a Mediterranean setting

**DOI:** 10.1002/brb3.2440

**Published:** 2021-12-15

**Authors:** Alejandro Garcia‐Rudolph, Joan Sauri, Alberto Garcia‐Molina, Blanca Cegarra, Eloy Opisso, Josep Maria Tormos, Dietmar Frey, Vince Istvan Madai, Montserrat Bernabeu

**Affiliations:** ^1^ Universitat Autònoma de Barcelona Bellaterra Cerdanyola del Vallès Spain; ^2^ Fundació Institut d'Investigació en Ciències de la Salut Germans Trias i Pujol Badalona Spain; ^3^ Institut Guttmann Hospital de Neurorehabilitacio Badalona Spain; ^4^ CLAIM Charité Lab for AI in Medicine Charité Universitätsmedizin Berlin Berlin Germany; ^5^ QUEST Center for Transforming Biomedical Research Berlin Institute of Health (BIH), Berlin, Germany, Charité – Universitätsmedizin Berlin Berlin Germany; ^6^ School of Computing and Digital Technology Faculty of Computing, Engineering and the Built Environment, Birmingham City University United Kingdom

**Keywords:** behavioral, COVID‐19, emotional, informal caregivers, stress, stroke, traumatic brain injury

## Abstract

**Introduction:**

Even in nonpandemic times, persons with disabilities experience emotional and behavioral disturbances which are distressing for them and for their close persons. We aimed at comparing the levels of stress in emotional and behavioral aspects, before and during coronavirus disease 2019 (COVID‐19), as reported by informal family caregivers of individuals with chronic traumatic brain injury (TBI) or stroke living in the community, considering two different stratifications of the recipients of care (cause and injury severity).

**Methods:**

We conducted a STROBE‐compliant prospective observational study analyzing informal caregivers of individuals with stroke (IC‐STROKE) or traumatic brain injury (IC‐TBI).

IC‐STROKE and IC‐TBI were assessed in‐person before and during COVID‐19 online, using the Head Injury Behavior Scale (HIBS). The HIBS comprises behavioral and emotional subtotals (10 items each) and a total‐HIBS. Comparisons were performed using the McNemar's test, Wilcoxon signed‐rank test or *t*‐test. Recipients of care were stratified according to their injury severity using the National Institutes of Health Stroke Scale (NIHSS) and the Glasgow Coma Scale (GCS).

**Results:**

One hundred twenty‐two informal caregivers (62.3% IC‐STROKE and 37.7% IC‐TBI) were assessed online between June 2020 and April 2021 and compared to their own assessments performed in‐person 1.74 ± 0.88 years before the COVID‐19 lockdown.

IC‐STROKE significantly increased their level of stress during COVID‐19 in five emotional items (impatience, frequent complaining, often disputes topics, mood change and overly sensitive) and in one behavioral item (overly dependent). IC‐TBI stress level only increased in one behavioral item (impulsivity).

By injury severity, (i) mild (14.7%) showed no significant differences in emotional and behavioral either total‐HIBS (ii) moderate (28.7%) showed significant emotional differences in two items (frequent complaining and mood change) and (iii) severe (56.6%) showed significant differences in emotional (often disputes topics) and behavioral (impulsivity) items.

**Conclusions:**

Our results suggest specific items in which informal caregivers could be supported considering cause or severity of the recipients of care.

## Graphical abstract

We reported results of the coronavirus disease 2019 (COVID‐19) impact from a psychological perspective, considering 10 emotional and 10 behavioral items, using the Head Injury Behavior Scale (HIBS) on informal family caregivers (*n* = 122) of individuals with stroke or traumatic brain injury (TBI) living in the community. We identified five specific emotional items and one behavioral item which were sources of increased stress to informal caregivers of individuals with stroke, while informal caregivers of individuals with TBI were impacted only in one (behavioral) item, suggesting a masking effect on them due to pre‐existing stress. Such identified items may be taken as an initial step to developing personalized therapeutic interventions provided by clinicians (e.g., as online remote services) to informal caregivers.

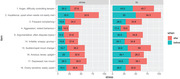

## 1 INTRODUCTION

Informal care is considered a cornerstone of all long‐term care provision systems in Europe (EU, 2018). It is also gaining increasing recognition in international policy circles as a key issue for future welfare policy. Estimates suggest that as much as 80% of all long‐term care in Europe is provided by informal carers (Frontiera et al., 2019). In the United States, approximately 43.5 million people are caregivers to a family member with a disability or illness (Gottschalk et al., 2020).

Caregiving can affect a caregiver's emotional well‐being and their social activities as they often feel that they are not prepared for their role due to a wide variety of personal factors and lack of skills (Woodford et al., 2018).

A first step to developing therapeutic interventions provided by clinicians targeting caregiver preparedness is to better understand factors which influence such burden levels. This might enable the development of effective interventions to prepare and support caregivers in their role (Lieshout et al., 2020). A recent thematic analysis reporting on informal caregivers of community dwelling people with traumatic brain injury (TBI) highlighted the health systems’ reliance on informal care and the importance of supporting them: Healthcare professionals need to consider and respond to the impact that changing circumstances have on the capacity of informal caregivers to manage their workload (McIntyre et al., 2018).

The coronavirus disease 2019 (COVID‐19) began spreading in China at the end of 2019 and, to date (October 2021) represents an international health emergency without precedents in terms of its health, economic and organizational effects on people's lives (WHO, 2021).

Spain has been one of the most affected countries in the world in terms of relative and absolute number of diagnosed cases. On March 13, 2020 (legally effective on March 15), the government declared a national state of alarm, with regulations targeted to facilitate diagnosis, ensure appropriate treatment of cases and reduce the spread of COVID‐19, including measures of national lockdown, confinement of the population and restricted mobility (Laxe et al., 2020). While these experiences have been felt globally, the COVID‐19 pandemic has introduced additional vulnerability and marginalization to those with some type of functional impairment—people with disabilities, chronic illness or frailty due to aging (Kang et al., 2020).

Evidence suggests, even in nonpandemic times, that persons with disabilities experience emotional and behavioral disturbances which are distressing for both individuals with disabilities and their close persons (Kim, 2007). There are limited data on the impact or added burden of a pandemic (or other calamities) on informal caregivers of persons with TBI or stroke.

TBI is a growing public health concern and represents the greatest contributor to disability globally among all trauma‐related injuries (Dewan et al., 2018). There is consensus over the fact that the disability associated with TBI stems mainly from the cognitive, emotional, and behavioral alterations that complicate its course (Jorege et al., 2015). Furthermore, it has been recognized that these disturbances tend to be chronic, difficult to treat and, occasionally, progressive (Bavisetty et al., 2008).

Stroke is a major cause of long‐term disability worldwide with increasing number of young people being affected by stroke in low‐ and middle‐income countries (Katan et al., 2018). Behavioral disturbances have been reported as frequent complications in stroke survivors (Ferro et al., 2009). As reported in previous research, survivors of TBI very often experience symptoms ranging from irritability to aggressive outbursts (Dyer et al., 2006).

Previous research remarked that increased severity of acquired injury is related to higher levels of caregiver stress. For example, Doser and Norub (2016) demonstrated over a long period (from 3 to 6 years) that the main factor influencing the burden of spouse‐caregivers was the severity of the injury of the patients. Considering a shorter period after injury (2 years) Laratta et al. (2020) recently identified injury severity being associated with a higher level of spouse‐caregiver burden. Similarly, the CONOCES study concluded that both the burden borne by informal caregivers and the likelihood of them being at a high risk of burnout (at 3 and 12 months post event) were associated with the severity of the stroke (Oliva Moreno et al., 2018). Meanwhile complaints on behavioral issues (such as low self‐activation, lack of initiative or apathy) have been previously reported in survivors of stroke (Caeiro et al., 2013).

Consequently, we hypothesized that during COVID‐19, specific emotional (e.g., anger, irritability and impatience) or behavioral (e.g., lack of initiative and overly dependency) aspects could be identified as sources of increased stress to caregivers when recipients of care are stratified by cause or by injury severity. Such aspects may be taken as an initial step for developing personalized therapeutic interventions provided by clinical professionals (e.g., as online remote services) to informal caregivers.

Therefore, this study aims at comparing the levels of stress in a set of 10 emotional and 10 behavioral standardized items, as reported by informal family caregivers of people with chronic TBI or stroke living in the community before and during the outbreak of COVID‐19. We stratified the participants in (i) informal caregivers of people with TBI (IC‐TBI) and informal caregivers of people with stroke (IC‐STROKE) and (ii) informal caregivers of mild, moderate or severe recipients of care.

## 2 MATERIALS AND METHODS

### 2.1 Study design

We conducted a prospective observational study analyzing informal caregivers of individuals with acquired brain injury (TBI or stroke) at chronic phase, with more than 3 years since injury, who were living in the community. The individuals with stroke or TBI were selected from the electronical health records of Institut Guttmann‐ Neurorehabilitation hospital in Catalonia, Spain.

Before COVID‐19, it was usual practice for patients and their informal caregivers to periodically (approximately every 18 months) visit the hospital. Such in‐person follow‐up visits aimed to assess their medical and psychosocial status. During COVID‐19, in‐person follow‐up visits were suspended and online assessments were implemented as part of the hospital's remote services.

Therefore, only informal caregivers of individuals with stroke or TBI registered in the hospital's electronical health records with at least one in‐person visit to the hospital (before the COVID‐19 outbreak) and who responded to the online assessment (during COVID‐ 19) were included in this study. Recruitment period for the online assessment was between June 2020 and April 2021. This study conforms to the Strengthening the Reporting of Observational Studies in Epidemiology (STROBE) Guidelines (STROBE, 2021).

### 2.2 Participants

Eligible participants were informal caregivers of individuals with the diagnosis of stroke or TBI (at the moment of injury were aged ≥18), living in the community with electronical health records including complete data.

Individuals with stroke or TBI were excluded for the following reasons: diagnosis of concomitant comorbidity (e.g., spinal cord injury, brain tumors and anoxia) and a previous history of another disabling condition or not living in the community.

To satisfy the informal caregiver criteria, the participant had to be a first‐degree relative (parent, sibling or spouse) who had spent at least 12 months looking after the person with TBI or stroke and who had the following characteristics: (i) is responsible for and makes decisions concerning the care of the person with TBI or stroke, regardless of whether he/she lives with the latter and/or (ii) spends most of the time, daily or weekly, accompanying/looking after the person with TBI or stroke. The informal caregiver might look after no more than one dependent person (De Arroyabe et al., 2013).

Every eligible participant was contacted as part of the routine clinical follow‐up. Consequently, participants completed the online measures analyzed in this study as part of a virtual visit involving other assessments, not analyzed in this study. Such assessments include, for example the Patient Competency Rating Scale (PCRS) to assess self‐awareness following brain injury.

Informal caregivers answered their follow‐up online assessments within 10 days since contacted; therefore, all assessments were completed between June 2020 and April 2021.

### 2.3 Remote follow‐up service and the Head Injury Behavior Scale

The online assessments were implemented during the COVID‐19 lockdown in order to provide a remote follow‐up service. Each participant received the online assessments by an SMS message sent to the participant's mobile phone. This SMS was sent by the professional from the hospital's Psychosocial Unit in charge of the online follow‐up. The online assessments include the same questionnaires and measurements as when participants were assessed (before the COVID‐19 outbreak) in‐person during their follow‐up visits.

In this study, we focused on one of such measurements: the Head Injury Behavior Scale (HIBS) (Godfrey et al., 2003). The Psychosocial Unit of the hospital routinely performs HIBS follow‐up every 3 years.

The HIBS was implemented to assess distress in caregivers of persons with acquired brain injury. It is a 20‐item scale describing common psychological problems that occur following brain injury. For each of the items in the scale, the caregiver is asked, “Is the behavior a problem?” (yes/no) and “How much distress does this problem cause?.” Distress ratings are recorded for the identified items using a four‐point scale: 1 (no distress); 2 (mild distress); 3 (moderate distress) and 4 (severe distress). The HIBS includes two subscales: Emotional Regulation and Behavioral Regulation. The Emotional Regulation subscale assesses behaviors reflecting impaired emotional control and occurring during interactions with caregivers (e.g., sudden/rapid mood changes, depression, anger and aggression). The Behavioral Regulation subscale assesses problems that carry less emotional valence for caregivers (e.g., lack of control over behavior, inappropriate behavior for social situations, lack of motivation, or lack of interest in doing things) (Godfrey et al., 2003).

The HIBS has been previously used in assessing emotional and behavioral problems in individuals with chronic TBI (Villalobos et al., 2020) (Marsh et al., 2006) or stroke (Orfei et al., 2009). In this study, we used the version of the HIBS adapted to the Spanish language (De Arroyabe et al., 2013). A description of the 20 HIBS items is presented in Table S1.

### 2.4 Clinical and demographic variables

Demographics (age, sex, years of education and marital status), as well as time since onset of the injury, were collected. Stroke severity of the injury was assessed within 24 h before acute phase discharge by the National Institutes of Health Stroke Scale (NIHSS) and stratified as follows: mild (1–4), moderate (NIHSS 5–14) or severe (NIHSS ≥15) (Williams et al., 2000).

The Glasgow Coma Scale (GCS) (Teasdale et al., 1974) was used to assess TBI severity at admission and was stratified as mild (14–15), moderate (9–13) or severe (3‐8) (Mena et al., 2011). All data were collected from the hospital’ electronical health records of the individuals with TBI or stroke.

### 2.5 Statistical analyses

All statistical analyses were performed in R‐v3.6.3 (64 bits) (The R project, 2021), and level of significance was set at *p* = .05. Descriptive statistics were used for demographic and clinical characteristics of individuals with stroke or TBI. Responses to HIBS were compared before and during the COVID‐19 outbreak and during it using the McNemar's test for nominal data (2 × 2 contingency tables with a dichotomous trait, caused stress [yes, no]), the Wilcoxon ranked test or paired *t*‐test when appropriate (HIBS total, emotional and behavior subtotals). The Shapiro Wilk test was used to assess normality, Levene test for homogeneity of variances and Cohen's *d* to assess effects sizes (small effect size [*d* = 0.1], medium [*d* = 0.3] and large effect size [*d* = 0.5]).

### 2.6 Ethical considerations

The study follows the Declaration of Helsinki and was approved by the Ethics Committee of Clinical Research of Institut Guttmann‐ Neurorehabilitation hospital. The participants were anonymized and nonidentifiable.

## 3 RESULTS

The initial number of eligible participants, considering the criteria described in Section 2.2. was *n* = 191, four of them had difficulties in understanding or expressing in the Spanish language as detected by members of the research team, which prevented him/her from being able to collaborate. One hundred and eighty‐seven participants received the SMS in their mobile phones, 24 (12.8%) were not a first‐degree relative (parent, sibling or spouse) who had spent at least 12 months looking after the person with TBI or stroke, 3 (1.6%) were looking after more than one dependent person, 11 (5.8%) did not have an available Functional Independence Measure (FIM) in person assessment and 27 (14.4%) did not complete the online HIBS assessment.

Consequently, a total of 122 informal caregivers participated in the study, 76 (62.3%) were IC‐STROKE and 46 (37.7%) were IC‐TBI.

Table 1 presents the demographics and clinical characteristics of the individuals with TBI and stroke. The mean age at the moment of online assessment was 55 (11) years, and 70% of the individuals were men.

**TABLE 1:** Demographics and clinical characteristics of all individuals with stroke (*n* = 76) or traumatic brain injury (*n* = 46) included in the study

Variables	Total participants (N = 122)
Sex (%)	
Male	71.3
Female	28.7
Age at the moment of the online assessment, mean (SD)	55.49 (11.82)
Age <65 at the moment of the online assessment (%)	78.7
Age ranges at the moment of the online assessment (%)	
18–30	1.6
31–45	24.6
46–60	38.5
61–75	32.0
76+	3.3
Time (in days) since lockdown (March 14) to the online assessment, mean (SD)	191 (73)
Time (in years) since closest in‐person assessment to lockdown (March 14), mean (SD)	1.74 (0.88)
Time (in years) since injury to online assessment, mean (SD)	8.47 (7.48)
Age at the moment of injury in years, mean (SD)	44.74 (14.47)
Injury origin, *n* (%)	
Traumatic brain injury	46 (37.7%)
Stroke	76 (62.3%)
Type of stroke, *n* (%)	
Hemorrhagic stroke	36 (29.5%)
Ischemic stroke	40 (32.8%)
Severity of stroke (NIHSS) (%)	
Mild	13.8
Moderate	43.0
Severe	43.2
Severity of traumatic brain injury (GCS) (%)	
Mild	15.6
Moderate	11.1
Severe	73.3
FIM in‐person assessment, mean (SD)	
Cognitive FIM	26.48 (8.82)
Motor FIM	61.32 (26.16)
Total FIM	87.81 (33.30)
Time in years since FIM in‐person assessment to the online assessment, mean (SD)	0.81 (1.31)
Years of education at the moment of the online assessment (%)	
Read and write (<2 years)	6.6
Primary (2–5 years)	38.5
Secondary (6–12 years)	29.5
Higher (>13 years)	25.4
Marital status. Married (%)	64.8
Location where respondents were living at the moment of answering the online assessment (%)	
Barcelona	72.8
Girona	10.7
Tarragona	9.9
Lérida	6.6

*Note*: All characteristics are presented as percentages (%), unless otherwise indicated.

Abbreviations: FIM, Functional Independence Measure; NIHSS, National institute of Health Stroke Scale; GCS, Glasgow Comma Scale.

In relation to the injury severity, 70% of the individuals with TBI were severe, and 86% of the individuals with stroke were moderate or severe. The mean time since lockdown (March 14) to the online assessment was 191 (73) days. The mean time since the closest in‐person assessment to lockdown (March 14) was 1.74 (0.88) years. The mean time since injury to online assessment was 8.47(7.48) years.

### 3.1 Stratification by IC‐TBI or IC‐STROKE

We then compared the emotional regulation subtotal, the behavioral regulation subtotal and the total HIBS score for IC‐TBI (N = 46, 37.7%) and IC‐STROKE (N = 76, 62.3%), as presented in Table 2.

**TABLE 2:** Paired comparisons for precoronavirus decease 2019 (COVID‐19) assessments and during COVID‐19 assessments for Head Injury Behavior Scale (HIBS) emotional, behavioral and total

Stratification	Subscale	COVID‐19	Median	Mean (SD)	SE Mean	CI mean 0.95%	Shapiro‐Wilk normality test (*p*)	Wilcoxon Signed rank test (W)	Wilcoxon Signed rank test (*p*)	Effect size (*d*)
All (N = 122)	Emotional	Before	5.50	8.09 (8.47)	0.77	1.52	<.0001	1801	**<.001**	−0.317
		During	9.00	11.25 (10.71)	0.97	1.92	<.0001			
	Behavioral	Before	6.00	8.47 (8.37)	0.76	1.50	<.0001	2060	**.004**	−0.256
		During	9.00	10.82 (9.44)	0.85	1.69	<.0001			
	Total	Before	13.50	16.56 (15.42)	1.40	2.76	<.0001	1853.5	**<.001**	−0.323
		During	18.00	22.07 (18.63)	1.69	3.34	<.0001			
IC‐TBI (N = 46)	Emotional	Before	9.00	10.00 (9.30)	1.37	2.76	<.0001	340	.109	−0.236
		During	9.50	12.02 (10.36)	1.53	3.08	<.0001			
	Behavioral	Before	8.00	9.91 (8.20)	1.21	2.44	<.0001	305.5	**.027**	−0.325
		During	11.50	12.85 (9.40)	1.39	2.79	<.0001			
	Total	Before	17.00	19.91 (15.79)	2.33	4.69	<.0001	305.5	**.027**	−0.325
		During	21.50	24.87 (17.65)	2.60	5.24	<.0001			
IC‐STROKE (N = 76)	Emotional	Before	4.00	6.93 (7.76)	0.89	1.77	<.0001	584.5	**.001**	−0.365
		During	7.00	10.79 (10.97)	1.26	2.51	<.0001			
	Behavioral	Before	5.00	7.59 (8.40)	0.96	1.92	<.0001	797	.072	−0.206
		During	8.00	9.59 (9.32)	1.07	2.13	<.0001			
	Total	Before	9.00	14.53 (14.92)	1.71	3.41	<.0001	663	**.004**	−0.323
		During	15.00	20.38 (19.11)	2.19	4.37	<.0001			

Abbreviations: IC‐TBI, informal caregivers of people with TBI; IC‐STROKE, informal caregivers of people with stroke.

When considering emotional regulation subtotals, no significant differences were found in IC‐TBI (*p* = .109), but differences were significant for IC‐STROKE (*p* = .001) and with the highest effect size *d* = −0.365

Meanwhile, the opposed is observed when considering behavioral regulation subtotals, no significant differences were found in IC‐STROKE (*p* = .072), but differences were significant for IC‐TBI (*p* = .027).

We then compared individuals with stroke with individuals with TBI considering demographics and clinical variables, as presented in Table 3.

**TABLE 3:** Comparison of demographics and clinical characteristics of individuals with stroke (*n* = 76) and traumatic brain injury (*n* = 46)

Variables	Stroke (N = 76)	TBI (N = 46)	*p*
Sex (%)			.935
Male	71.1	71.7	
Female	28.9	28.3	
Age at the moment of the online assessment, mean (SD)	59.73 (9.03)	48.49 (12.62)	**<.001**
Age >65 at the moment of the online assessment (%)	26.3	13.0	.083
Age ranges at the moment of the online assessment (%)			**<.001**
18–30	0	4.3	
31–45	9.2	50.0	
46–60	47.4	23.9	
61–75	39.5	19.6	
76+	3.9	2.2	
Time (in days) since lockdown (March 14) to the online assessment, mean (SD)	200 (71)	178 (73)	.120
Time (in years) since closest in‐person assessment to lockdown (March 14), mean (SD)	1.73 (0.92)	1.76 (0.83)	.334
Time (in years) since injury to online assessment, mean (SD)	6.74 (7.30)	11.34 (6.93)	**<.001**
Age at the moment of injury in years, mean (SD)	50.71 (9.25)	34.89 (16.16)	**<.001**
Severity (%)			.642
Mild	13.8	15.6	
Moderately severe and severe	86.2	84.4	
FIM in‐person assessment, mean (SD)			
Cognitive FIM	28.13 (8.04)	23.76 (9.45)	**.008**
Motor FIM	63.50 (23.01)	57.73 (30.61)	.719
Total FIM	91.63 (29.09)	81.50 (38.80)	.378
Time in years since FIM in‐person assessment to the online assessment, mean (SD)	0.80 (1.20)	0.82 (1.50)	.395
Years of education at the moment of online assessment (%)			.130
Read and write (<2 years)	9.2	2.2	
Primary (2–5 years)	35.5	43.5	
Secondary (6–12 years)	34.2	21.7	
Higher (>13 years)	21.1	32.6	
Marital status. Married (%)	81.6	37.0	**<.001**
Support in ADLs			.210
Daily: 24 h	32.3	35.6	
Daily: 12–23 h	6.5	11.1	
Daily:6 – 11 h	8.1	2.2	
Daily: 3–5 h	19.4	6.7	
Daily: less than 3 h	14.5	15.6	
Occasional	6.5	17.8	
Not needed	12.9	11.1	
Location where informal caregivers were living at the moment of answering the online assessment (%)			.483
Barcelona	73.3	71.7	
Girona	13.3	6.5	
Tarragona	8.0	13.0	
Lérida	5.3	8.7	

All characteristics are presented as percentages (%), unless otherwise indicated. Bold values are for *p* < 0.05.

Abbreviations: ADLs: Activities of Daily Living; FIM, Functional Independence Measure; TBI, traumatic brain injury

Significant differences were found in age at the moment of online assessment. Individuals with TBI were significantly younger (*p* < .001) with 50% of them between 31 and 45 years old at the moment of the online assessment, whereas only 9.2% of the individuals with stroke were between 31 and 45 years old at the moment of online assessment (*p* < .001).

When comparing functional independence, as assessed using the FIM, cognitive FIM was significantly higher in individuals with stroke (*p* = .008).

We further compared each of the 20 HIBS items separately for IC‐STROKE and IC‐TBI, before and during COVID‐19, in order to identify specific items causing (mild, moderate or severe) stress, as detailed in Table 4. For IC‐TBI only in one of them (item 5: Impulsivity; does things without thinking), a significant increase of the level of stress was reported: 32.6% of the informal caregivers had stress due to this item before and during COVID‐19, and the percentage of informal caregivers who had stress due to this item significantly increased to 52.2%, as detailed in Table 4.

**TABLE 4:** Head Injury Behavior Scale (HIBS) items comparison (McMenar test) before and during coronavirus disease 2019 (COVID‐19) as reported by informal caregivers of people with stroke (IC‐STROKE; *n* = 76) and informal caregivers of people with TBI (IC‐TBI; *n* = 46) when answering the question “does this item cause you mild, moderate or severe stress?”

		IC‐STROKE (N = 76)			IC‐TBI (N = 46)		
		Before	During		Before	During	
Id	Item	Yes (%)	Yes (%)	*p*	Yes (%)	Yes (%)	*p*
1	Anger; difficulty in controlling temper	26.3	27.6	.998	43.5	45.7	.998
2	Impatience; upset when needs not easily met	**35.5**	**52.6**	**.020**	50.0	54.3	.772
3	Frequent complaining	**15.8**	**34.2**	**.002**	28.3	34.8	.546
4	Aggression; violent behavior	9.2	14.5	.342	17.4	19.6	.998
6	Argumentative; often disputes topics	**13.2**	**31.6**	**.005**	30.4	34.8	.789
14	Irritable; snappy; grumpy	15.8	27.6	.0664	26.1	34.8	.386
15	Sudden/rapid mood change	**19.7**	**38.2**	**.005**	32.6	50.0	.061
16	Anxious; tensed; uptight	25.0	32.9	.326	39.1	28.3	.301
17	Depressed; low mood	26.3	35.5	.190	34.8	39.1	.813
19	Overly sensitive; easily upset	**18.4**	**36.8**	**.014**	28.3	32.6	.789
5	Impulsivity; does things without thinking	19.7	30.3	.080	**32.6**	**52.2**	**.038**
7	Lacks control over behavior; behavior is inappropriate for social situations	18.4	25.0	.332	19.6	34.8	.096
8	Overly dependent; relies on others unnecessarily	**25.0**	**38.2**	**.033**	30.4	47.8	.135
9	Poor decision making; does not think of consequences	27.6	31.6	.662	32.6	45.7	.286
10	Childish; at times behavior is immature	30.3	35.5	.540	23.9	39.1	.145
11	Poor insight; refuses to admit difficulties	25.0	30.3	.522	32.6	39.1	.605
12	Difficulty in becoming interested in things	13.2	22.4	.121	23.9	34.8	.301
13	Lack of initiative; does not think for him/herself	18.4	28.9	.135	32.6	34.8	.998
18	Irresponsible; cannot always be trusted	19.7	25.0	.479	28.3	39.1	.301
20	Lacks motivation; lack of interest in doing things	28.9	31.6	.850	32.6	23.9	.479

*Note*: HIBS‐emotional items: Id = 1, 2, 3, 4, 6, 14, 15, 16, 17, 19, HIBS‐behavioral items: Id = 5, 7, 8, 9, 10, 11, 12, 13, 18, 20.

Meanwhile, for IC‐STROKE, significant differences were reported in six items: impatience, frequent complaining, often disputes topics, mood change, overly sensitive and overly dependent.

**FIGURE 1:**
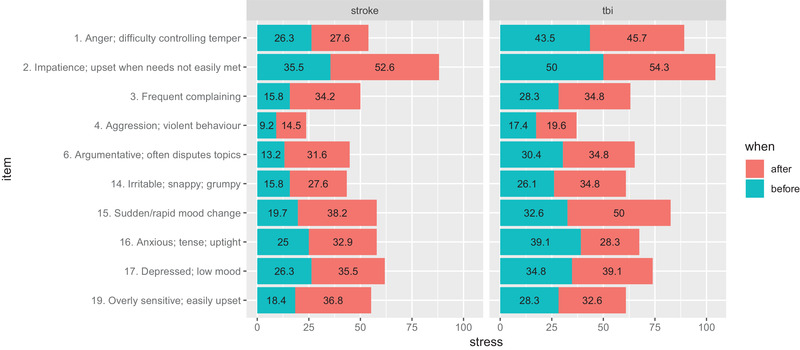
Head Injury Behavior Scale (HIBS)‐emotional items’ comparison before and during coronavirus decease 2019 (COVID‐19) as reported by informal caregivers of individuals with stroke (*n* = 76) and individuals with traumatic brain injury (TBI; n = 46)

A graphical representation of Table 4 (considering only the emotional regulation items) is presented in Figure 1.

### 3.2 Stratification by injury severity

We then compared the emotional regulation subtotal, the behavioral regulation subtotal and the total HIBS for informal caregivers of the recipients of care, now stratified according to their injury severity (as defined in Section 2.4).

When considering individuals with mild injury (N = 18, 14.7%), no significant differences were found in the emotional regulation subtotal (*p* = .273), and the behavioral regulation subtotal (*p* = .09) either the total HIBS (*p* = .23).

When considering individuals with moderate injury (N = 35, 28.7%), we found significant differences in the emotional regulation subtotal (*p* = .04). No significant differences were found in the behavioral regulation subtotal (*p* = .62) either the total HIBS (*p* = .18).

Finally, when considering individuals with severe injury (N = 69, 56.6%), we found significant differences in all three of them: the emotional regulation subtotal (*p* = .03), the behavioral regulation subtotal (*p* = .02) and the total HIBS (*p* = .01), as presented in Table 5.

**TABLE 5:** Paired comparisons for precoronavirus disease 2019 (COVID‐19) assessments and during COVID‐19 assessments for Head Injury Behavior Scale (HIBS) emotional, behavioral and total stratified by injury severity

Stratification	Dimension	COVID‐19	Median	Mean (SD)	SE Mean	CI mean 0.95%	Shapiro‐Wilk normality test (*p*)	Wilcoxon Signed rank test (W) or *t*‐test	Wilcoxon Signed rank test or *t*‐test (*p*)	Effect size (*d*)
Mild (N = 18)	Emotional	Before	6.00	7.73 (6.77)	1.74	3.75	.15	−1.13	.273	−0.18
		During	7.00	11.20 (12.55)	3.24	6.95	.01			
	Behavioral	Before	6.00	7.40 (7.22)	1.86	4.00	.04	21.5	.09	−0.19
		During	9.00	10.13 (8.91)	2.30	4.93	.04			
	Total	Before	14.00	15.13 (13.01)	3.36	7.20	.22	−1.24	.23	−0.19
		During	15.00	21.33 (20.91)	5.39	11.57	0.05			
Moderate (N = 35)	Emotional	Before	5.00	7.86 (7.78)	1.42	2.90	<.001	**112.5**	**.04**	−**0.37**
		During	10.00	12.43 (10.98)	2.01	4.10	.01			
	Behavioral	Before	5.00	7.17 (8.28)	1.51	3.09	<.001	168	.62	−0.10
		During	6.00	9.23 (9.62)	1.76	3.59	.001			
	Total	Before	9.50	15.03 (15.37)	2.81	5.74	<.001	133	.18	−0.24
		During	17.00	21.66 (19.32)	3.53	7.21	.002			
Severe (N = 69)	Emotional	Before	5.50	8.07 (9.39)	1.23	2.47	<.001	**480.5**	**.03**	−**0.28**
		During	7.00	10.36 (9.76)	1.28	2.57	<.001			
	Behavioral	Before	6.00	8.56 (8.32)	1.09	2.19	<.001	**461.5**	**.02**	−**0.29**
		During	9.50	11.01 (9.33)	1.22	2.45	<.001			
	Total	Before	13.50	16.64 (15.79)	2.07	4.15	<.001	**457.5**	**.01**	−**0.32**
		During	18.50	21.38 (16.95)	2.23	4.46	.001			

**FIGURE 2:**
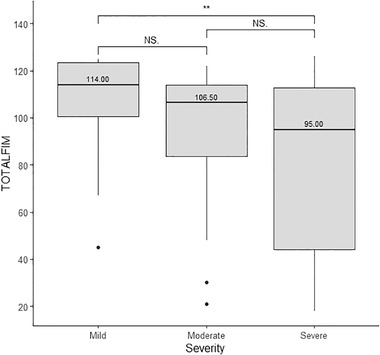
Total Functional Independence Measure (FIM) boxplots stratified by injury severity levels

Injury severity was assessed within 24 h before acute phase discharge, and the mean length of stay in acute phase was 21.3 (8.4) days. Figure 2 presents the total FIM scores boxplots for each severity level, showing that individuals with higher severity still get lower FIM scores despite the FIM scores were assessed several years later than the injury severity assessments. As presented in Table 1, the time (in years) since injury to the online assessment was 8.47 (7.48) years, whereas the time in years since the FIM in‐person assessment to the online assessment was 0.81 (1.31) years.

As shown in Figure 2, individuals with mild severity get significantly higher total FIM scores when compared with individuals with severe injury, and this is represented with two asterisks in Figure 2. Differences in total FIM scores are not significant when comparing mild with moderate and moderate with severe. Nevertheless, Figure 2 clearly shows that as severity increases, the FIM score decreases.

We further compared each of the 20 HIBS items separately for moderate and severe recipients of care, before and during COVID‐19, in order to identify specific items causing (mild, moderate or severe) stress, as detailed in Table 6.

**TABLE 6:** Head Injury Behavior Scale (HIBS) items comparison (McMenar test) before and during coronavirus disease 2019 (COVID‐19) as reported by informal caregivers for moderate (*n* = 35) and severe (*n* = 69) recipients of care when answering the question “does this item cause you mild, moderate or severe stress?”

		Moderate (N = 35)			Severe (N = 69)		
		Before	During		Before	During	
Id	Item	Yes (%)	Yes (%)	*p*	Yes (%)	Yes (%)	*p*
1	Anger; difficulty controlling temper	30.0	40.0	.505	34.5	34.5	1.00
2	Impatience; upset when needs not easily met	43.3	60.0	.227	37.9	50.0	.121
3	Frequent complaining	**20.0**	**43.3**	**.045**	18.9	27.6	.182
4	Aggression; violent behavior	10.0	13.3	.998	12.1	15.5	.683
6	Argumentative; often disputes topics	23.3	33.3	.546	**15.5**	**32.8**	**.03**
14	Irritable; snappy; grumpy	20.0	36.6	.182	17.2	27.6	.181
15	Sudden/rapid mood change	**26.7**	**56.7**	**.026**	27.6	36.2	.267
16	Anxious; tensed; uptight	33.3	43.3	.579	31.0	27.6	.802
17	Depressed; low mood	33.3	43.3	.579	31.0	34.5	.823
19	Overly sensitive; easily upset	26.7	33.3	.772	22.4	31.0	.332
5	Impulsivity; does things without thinking	30.0	33.3	.998	**17.2**	**37.9**	**.005**
7	Lacks control over behavior; behavior is inappropriate for social situations	13.3	23.3	.505	17.2	27.6	.181
8	Overly dependent; relies on others unnecessarily;	23.3	40.0	.130	34.5	50.0	.095
9	Poor decision making; does not think of consequences	23.3	33.3	.449	24.1	39.6	.109
10	Childish; at times behavior is immature	23.3	30.0	.723	29.3	37.9	.358
11	Poor insight; refuses to admit difficulties	23.3	26.7	.998	27.5	34.4	.453
12	Difficulty in becoming interested in things	13.3	26.7	.220	20.7	25.9	.627
13	Lack of initiative; does not think for him/herself	13.3	30.0	.182	29.3	32.7	.823
18	Irresponsible; cannot always be trusted	23.3	26.7	.998	17.2	27.6	.181
20	Lacks motivation; lack of interest in doing things	30.0	33.3	.998	27.6	32.7	.662

*Note*: HIBS‐emotional items: Id = 1, 2, 3, 4, 6, 14, 15, 16,17 ,19, HIBS‐behavioral items: Id = 5, 7, 8, 9, 10, 11, 12, 13, 18, 20.

For recipients of care with moderate injury, a significant increase of the level of stress was reported by the informal caregivers in two emotional items (frequent complaining and sudden/rapid mood change). For severe recipients of care, a significant increase of the level of stress was reported in two items, one emotional (argumentative; often disputes topics) and the other behavioral (impulsivity; does things without thinking).

We did not include recipients of care with mild injury severity in Table 6 because (as reported in Table 5) no significant differences were found for them in the emotional regulation subtotal and the behavioral regulation subtotal either the total HIBS. Besides, only 14.7% of all recipients of care presented mild injury severity.

## 4 DISCUSSION

We studied the impact of COVID‐19 from a psychological perspective (using the HIBS 10 emotional and 10 behavioral items), on informal caregivers of individuals living in the community with chronic TBI or stroke, stratified by cause of injury and by injury severity of the recipients of care.

A recent survey conducted in Nashville, United States, by Morrow et al. (2021) on 47 individuals in the chronic phase of moderate‐severe TBI reported that for 51.06% of the participants, the pandemic has affected their sense of mental and physical well‐being. In our case, we analyzed 20 psychological items and only in one of them a significant increase of the level of stress was reported (Impulsivity; does things without thinking). Meanwhile as detailed in Table 4, in caregivers of people with stroke, significant differences were reported in six items: impatience, frequent complaining, often disputes topics, mood change, overly sensitive and overly dependent. Prepandemic studies among family caregivers of community dwelling stroke survivors report that informal caregivers’ total burden score was largely driven by their feelings of overly dependence (Morimoto et al., 2001), being dependency and immature behavior major causes of stress (Dankner et al., 2016).

Cohen et al. (2021) analyzed self‐reported changes in burden (CB) and intensity due to COVID‐19 (*n* = 835) of informal caregivers living in the United States. They found that men with higher initial levels of confidence interval were more likely to have an increase in CB due to the pandemic, but the association was not significant for women. There is no clear explanation for this finding although it may be due to gender differences in resilience among informal caregivers. Female caregivers may be more resilient than male caregivers (Gaugler et al., 2007), and this resilience may become magnified under periods of extreme stress and uncertainty, such as the COVID‐19 pandemic. In our case, as presented in Table 1, 71.3% of care recipients were males, suggesting that most of their informal caregivers could be females, though we did not collect demographic data on the informal caregivers. Nevertheless, it is known that women are more likely than men to be informal caregivers, among the whole population of informal caregivers themselves (Bauer et al., 2015). Cohen et al., concluded that a notable finding of their study was that higher initial CB was associated with a higher likelihood of increased CB during the COVID‐19 pandemic. This finding may be counterintuitive, as one could expect that there is a potential for a ceiling effect of CB—in other words, CB could not increase substantially due to the pandemic simply because it was already high (Hagell et al., 2017). One possible explanation for this finding is that the extra burdens of the pandemic, whether due to increased anxiety or other stressors, magnified the effects of caregiving on those with an already high level of CB. That was not the case in our IC‐TBI sample, a ceiling effect may be occurring for IC‐TBI, causing that an already high level of stress (as shown in Figure 1) masked the effect of the pandemic situation.

Seth et al. (2021) compared prepandemic (January 2020, *n* = 221) and early‐pandemic caregiving samples (April–June 2020, *n* = 177) in the United States and Latin America. They found that COVID‐19 did not seem to affect depression and self‐efficacy, nor did it affect participant's perception of their general overall health. The lack of impact of COVID‐19 on depression or overall health could either be due to the fact that the disease or threat of the disease does not appear to affect these parameters or that they were quite high to begin with and maybe had already reached a near‐ceiling effect. An important acknowledged limitation of the study was that authors were not able to determine whether the caregivers were living with the care recipients and if they were the sole caregivers of the care recipients.

Budnick et al. (2021) reported from an ad hoc survey (*n* = 1000) in Germany (April 21 to May 2, 2020) that informal caregivers during the COVID‐19 pandemic perceive additional burden.

Rodrigues et al. (2021) reported the impact of the pandemic on informal caregiving in Austria. They used a pre‐ and postonset of the pandemic research design based on a representative survey carried out in Austria in June 2020 (N = 2000) in combination with comparable 2015 data from the European Social Survey. Their findings suggest that both the prevalence and intensity of informal care remained stable after the onset of the first wave of the pandemic in Austria.

We were not able to find studies specifically addressing the impact of COVID‐19 on informal caregivers of people with TBI or stroke.

Pisano et al. (2020) compared 73 individuals with poststroke aphasia (PWA) in chronic phase and 81 matched healthy controls in Italy (Rome and Turin). The comparison between the two different time points (1 month before and 1 month after the COVID‐9 lockdown) led to a significant increase in depression and anxiety symptoms in both groups (PWA vs. control). Lower rates of depression and anxiety were found in PWA compared to the healthy group. Pisano et al. conclude that this evidence which, at first glance, seems to suggest that PWA have been partially spared from the impact of COVID‐19, actually masks a dramatic situation that has always characterized this population. Indeed, given that PWA already live in a state of social isolation and emotional instability, these conditions might have, paradoxically, limited the effects of the coronavirus (Pisano et al., 2020).

Our nonsignificant differences in the informal caregivers of individuals with TBI in 19 of the 20 items presented in Table 4 may be due to a similar masking effect, as shown in Figure 1 their stress levels were already high before the COVID‐19 outbreak. In relation to informal caregivers of individuals with stroke, we found significant differences in five out of the 10 HIBS emotional items, indicating a clear impact of the pandemic situation on them. Though, we did not stratify them considering informal caregivers of individuals with aphasia.

Injury severity has been reported to play a large role in the type of sequelae that result following TBI and thus influence the caregiver/family's function over time (Thompson et al., 2009). In our case, for mild injuries we identified no differences before and during COVID‐19. For moderate injuries, we identified differences in the total emotional regulation score and when injuries were severe, we identified differences in the three of the assessed totals. Therefore, our results suggest that for recipients of care with moderate injuries, support to informal caregivers should be focused on emotional aspects (mainly in frequent complaining and sudden/rapid mood change as shown in Table 6) rather than on behavioral. Meanwhile, for severe injuries, support should be focused on both emotional (specifically in argumentative; often disputes topics) and behavioral aspects (specifically in impulsivity; does things without thinking).

Acquired brain injury is considered a stressor that changes caregiver's psychosocial functioning (Perlesz et al., 2000). Higher distress and lower life satisfaction of caregivers are associated with low long‐term functional outcomes of persons with acquired brain injury, even after accounting for injury severity and cognitive functioning of care recipients (Vangel et al., 2011). As the person with acquired brain injury and his or her caregiver belongs to the same family system, both are influenced and influential agents (Vangel et al., 2011). Our findings are in line with such results; furthermore, in Table S3 we analyzed a stratification in two groups according to their FIM assessments (independent vs. dependent). The emotional, behavioral subtotals and total HIBS comparisons yielded that the informal caregivers to the dependent group presented higher effect sizes in all three of them.

Several limitations to this study are worth mentioning. First, the data were collected from informal caregivers of individuals living in the community but who had previously undertaken rehabilitation in one single tertiary center in Catalonia. As presented in Table 1, 72.8% of them were living in the province of Barcelona. Therefore, the generalization of these results should be considered carefully. Nevertheless, assessments by standardized tools (HIBS, FIM, NIHSS and GCS) allow for similar comparative studies, and the restricted physical locations allow for controlled variability in regional pandemic circumstances.

Second, the evolving nature of the pandemic may produce psychological effects, for example the COVID‐19 vaccination campaign started on December 27, 2020 in Spain (Vaccination Program, 2021). The initial phase of vaccination plan covered residents of senior homes and their carers and continued with healthcare workers and adults with major dependencies. Therefore, it did not involve any of the participants in this study, but it could somehow impact in their psychological status. Other contextual circumstances may have had an emotional impact (e.g., summer in Spain from June 2020 to September 2020).

Third, male gender accounts for about 70% of the individuals with stroke and individuals with TBI, suggesting a sex bias. Nevertheless, the proportion is similar to recent studies in similar settings (as presented in Supporting Information).

Fourth, demographic data (e.g., age, gender, level of education) were not collected on informal caregivers. The online assessments were implemented during the COVID‐19 lockdown in order to provide a remote follow‐up service, as performed in‐person before the COVID‐19 outbreak. Therefore, remote service included the same psychological assessments performed as in‐person visits, which did not collect demographic data on informal caregivers.

Fifth, online assessments have been criticized in previous studies. Several cons have been reported (Silva Durga, 2019): the researcher cannot determine questionnaire filling time and participants may abandon the survey giving partial data; if the participants have a doubt, researcher cannot clear it immediately. In our case, participants already knew the questionnaires, because they have already answered them during previous in‐person visits; therefore, cons were minimized. Furthermore, as remarked by Pisano et al. and Morrow et al., access to home‐based remote services is recommended during these pandemic times.

Future work includes (i) analyzing informal caregivers’ demographical variables, psychological status (e.g., HADS assessment) and burden (e.g., ZARIT assessment), (ii) conducting a specific analysis on caregivers of individuals with aphasia (approximately 40% of the hospital admissions) and (iii) in Table S2, we included a stratification by age in two groups (≤54 years (N = 55) and > 55 years (N = 67)) and found that differences were nonsignificant in the HIBS behavioral subtotal for the ≥55 years group, whereas in the younger group, differences were significant in both emotional and behavioral subtotals, leaving room for future analysis.

## 5 CONCLUSIONS

For the first time, we reported results of the COVID‐19 impact from a psychological perspective, considering 10 emotional and 10 behavioral items on informal family caregivers of individuals with stroke or TBI living in the community. We identified six specific HIBS items that have been significantly impacted by the COVID‐19 in informal caregivers of individuals with stroke. We found one item in caregivers of individuals with TBI, suggesting in their case a possible masking effect due to a pre‐existing situation of emotional instability.

When considering the severity of the recipients of care, we identified two emotional items for informal caregivers of moderate recipients of care and one emotional as well as one behavioral item for severe recipients of care. We identified a higher impact on emotional and behavioral subtotals as well as in total HIBS as severity of injury increased, further confirming them to be closely related to their long‐term functional independence outcomes

Our results can be used to suggest possible therapeutic interventions or support provided by clinicians to informal caregivers, specifically targeting the identified items or subtotals.

## FINANCIAL SUPPORT

This research was partially funded by H2020 Personalised Medicine by Predictive Modeling in Stroke for better Quality of Life (PRECISE4Q) Grant Nr 777107.

### PEER REVIEW

The peer review history for this article is available at https://publons.com/publon/10.1002/brb3.2440

## Data Availability

The data that support the findings of this study are available from the corresponding author upon reasonable request.
